# Enhancement of heat resistance of *Bacillus thermoamylovorans* drives enhanced PET degradation

**DOI:** 10.1016/j.engmic.2025.100256

**Published:** 2025-12-23

**Authors:** Yi-Mei Cai, Kang-Qi Hu, Xin-Yu Zhang, Hui Liu, Yan Huang, Wei Xia, Jing Wu, Zheng-Fei Yan

**Affiliations:** School of Biotechnology and Key Laboratory of Industrial Biotechnology Ministry of Education, Jiangnan University, 1800 Lihu Avenue, Wuxi 214122, China

**Keywords:** Thermotolerance, Heat shock protein, *Bacillus thermoamylovorans*, PET degradation, Protein synthesis

## Abstract

Microbial polyethylene terephthalate (PET) degradation has emerged as an environmentally friendly approach to reducing plastic pollution, but microbial activity is often limited by the high temperatures (60–70 °C) required for efficient degradation. In this study, six endogenous thermotolerant genes were identified in the PET-degrading strain *Bacillus thermoamylovorans* JQ3. Overexpression of the genes *hrcA, hsp20A,* and *hsp20B* significantly enhanced thermotolerance in *Escherichia coli*, which increased cell viability by >24.2% at temperatures above 40 °C. Similarly, overexpression of *hsp20A* in *B. thermoamylovorans* (*B. th*_Hsp20A) improved its thermotolerance, increasing cell viability by 46.8% at 50 °C and 69.8% at 60 °C. The *hsp20A*-encoded protein was identified as a small heat shock protein (sHSP). *B. th*_Hsp20A exhibited significantly improved PET degradation at 60 °C compared with 50 °C, which released 282 µg of terephthalic acid after 7 days of incubation, representing an 84.3% increase compared with the wildtype strain (153 µg). These results highlight that the overexpression of thermotolerant proteins, particularly sHSPs, significantly enhances microbial PET degradation under high-temperature conditions, offering a novel pathway for enhancing microbial PET waste degradation.

## Highlights

• First identification of thermotolerance genes in *Bacillus thermoamylovorans*.
• Small heat shock protein enhances cell survival under heat stress.
• Engineered stain achieves 84.3 % higher PET degradation at 60 °C than wild type.
• Improved PET degradation is attributed to enhanced protein stability and activity.

## Graphical abstract

**Figure:**
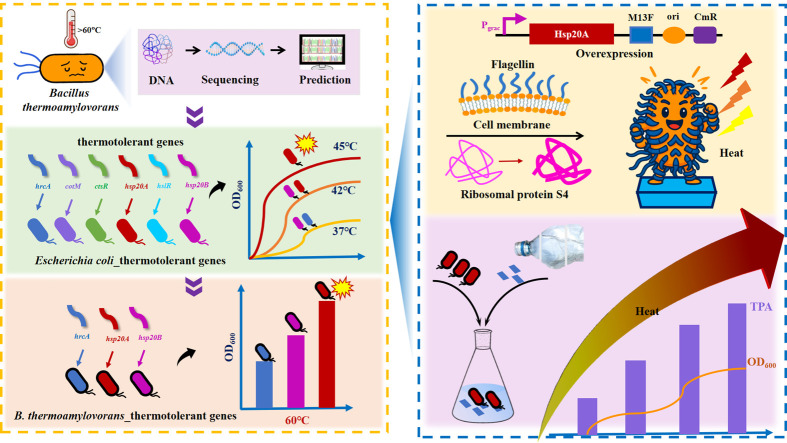

## 1 Introduction

Polyethylene terephthalate (PET) is a widely used polyester plastic, which is synthesized from terephthalic acid (TPA) and ethylene glycol (EG) [1,2]. Due to its durability, lightweight nature, and corrosion resistance, PET is widely used in textiles and packaging [3,4]. However, disposed PET waste poses significant environmental challenges. Traditional disposal methods, such as incineration and landfilling [5,6], are not only harmful to the environment but also to human health [7,8]. Microbial degradation of PET has gained attention as a more sustainable and eco-friendly alternative [[9], [10], [11]]. During microbial processing, reaction temperature is a critical factor in determining the final degradation rate of PET [12]. As the required temperature is close to the glass transition temperature (Tg) of PET (60–70 °C) [13,14], its amorphous regions become more flexible, thereby facilitating enzymatic attack [10,15].

Currently, most of the known PET-degrading strains are mesophilic, which exhibit optimal growth at 20–40 °C [16,17]. However, higher temperatures are often required for efficient PET degradation, which poses challenges for microbial growth. High temperatures harm microbial cells by inhibiting cell division and growth, disrupting cellular integrity, and compromising protein stability [18,19]. Thus, engineering PET-degrading strains with enhanced heat resistance is a promising strategy for improving the PET degradation efficiency.

Thermophiles have evolved to adapt to high-temperature environments to survive and thrive under heat stress. Their heat resistance is attributable to several mechanisms, including the presence of spore-forming genes, modification of their cell membrane composition, the production of thermophilic enzymes, and the upregulation of heat shock proteins (HSPs) [16,20,21]. HSPs (e.g., DnaK, GroEL, and GroES) play a critical role in maintaining protein stability by assisting in proper protein folding to counteract thermal stress [21,22]. The accumulation of HSPs is essential for microbial stress resistance and survival; therefore, reduced HSP levels impair both stress tolerance and normal cellular function [23,24].

A promising approach to enhance microbial heat resistance of mesophilic microbes is heterologous expression of thermotolerant genes from thermophiles in target microbes [[25], [26], [27]]. Liu et al. introduced HSP genes into *Saccharomyces cerevisiae*, which broadened its growth temperature range to 28–35 °C and reduced the fermentation time by 72 h [28]. Similarly, the overexpression of HSP genes from *Pyrococcus furiosus* in *Escherichia coli* significantly improved their heat resistance, increasing survival at 45 °C and 50 °C by 15.75- and 40.7-fold, respectively, while increasing ethanol production by 4.74-fold compared to the wild type strain [29].

In our previous work, *Bacillus thermoamylovorans* JQ3 was identified as a thermophile capable of utilizing PET as a carbon source [30] and has been deposited in the China Center for Type Culture Collection (CCTCC) under deposit number CCTCC M 20,211,351. The genome sequences of *B. thermoamylovorans* have been deposited in the NCBI database under the accession number SRR18149614. Here, we identified endogenous thermotolerant genes from *B. thermoamylovorans* and investigated their potential to enhance thermotolerance in *E. coli*. Subsequently, the optimal thermotolerant gene was overexpressed in *B. thermoamylovorans* to evaluate its effect on heat resistance and PET degradation.

## 2 Materials and methods

### 2.1 Materials

Amorphous PET (lcPET, product No ES301445, crystallinity:10 %) was obtained from Good-fellow Co., Ltd. (Bad Nauheim, Germany) and then micronized via liquid nitrogen grinding to yield powders with a particle size <500 µm. Bis(2-hydroxyethyl) terephthalate was purchased from Aladdin Industrial Co., Ltd. (Los Angeles, California, USA). Mono(2-hydroxyethyl) terephthalate (MHET) and TPA were supplied by Sigma-Aldrich (Saint Louis, Missouri, USA). All other chemicals were obtained from the Sinopec Group (Beijing, China).

### 2.2 Heat resistance analysis of wild-type *B. thermoamylovorans*

Single colonies were incubated in Luria-Bertani (LB) broth overnight at 50 °C and 200 rpm. The resulting culture was adjusted to an OD_600_ of 1.0 and transferred to 50 mL of LB broth at a 1 % inoculum. These cultures were incubated at 200 rpm and various temperatures (50, 60, and 70 °C). OD_600_ values were measured at 12-h intervals by using a P5 UV/Visible spectrophotometer (MAPADA, Shanghai, China). After 48 h of incubation, cells were collected and subjected to transmission electron microscopy (TEM) analysis.

### 2.3 Sequence analysis and structural prediction of the optimal HSP

The amino acid sequence of the optimal HSP was compared with sequences in GenBank, and a phylogenetic tree was constructed to analyze its relationship with other HSPs (Supplementary information 1). The phylogenetic trees were constructed using the neighbor-joining method in the MEGA 7.0 program with 2000 bootstrap replications. The secondary and tertiary structure predictions for the optimal HSP were performed using the Novopro (http://www.novopro.cn/), SOPMA (https://npsa.lyon.inserm.fr/cgi), and AlphaFold3 (https://alphafold3.org/) online platforms, respectively (Supplementary Information 2).

### 2.4 Plasmid construction and transformation

Plasmid construction and DNA manipulations were performed using standard molecular biology techniques. The candidate thermotolerant genes were amplified from the genome of *B. thermoamylovorans* and cloned into plasmids pET-24a(+) for *E. coli* and pHT01 for *B. thermoamylovorans*, respectively. Similarly, genes encoding flagellin and 30S ribosomal protein were cloned into the plasmid pHT01 (Fig. S1). *E. coli* JM109 was employed as a host strain for plasmid construction. The generated plasmids were transformed into *E. coli* BL21(DE3) and *B. thermoamylovorans*, referred to as *E. coli*_pET-24a(+) and *B. th*_pHT01, respectively. The recombinant strains were incubated in LB broth at 200 rpm or on solid LB agar plates (1.5 % w/v) at 37 °C for *E. coli* and 50 °C for *B. thermoamylovorans*. For selection, antibiotic pressure was applied as follows: 100 µg/mL ampicillin (Amp^R^) for *E. coli*_pHT01, 30 µg/mL kanamycin (Kan^R^) for *E. coli*_pET-24a(+), and 12.5 µg/mL chloramphenicol (Cm^R^) for *B. th*_pHT01. Positive colonies were confirmed via PCR using the primers detailed in Table S1, with the corresponding gene sequences listed in Table S2. A complete summary of all gene names, plasmids, and strains is provided in Table 1.

**Table 1:** Summary of recombinant plasmids and engineered strains.

Thermotolerant genes^a^	Expression vector^b^	Engineered strains^c^
/	pET24a(+)	*E. coli*_WT
*hrcA*	pET24a(+)_*hrcA*	*E. coli_*HrcA
*cotM*	pET24a(+)_*cotM*	*E. coli_*CotM
*ctsR*	pET24a(+)_*ctsR*	*E. coli_*CtsR
*hslR*	pET24a(+)_*hslR*	*E. coli_*HslR
*hsp20A*	pET24a(+)_*hsp20*A	*E. coli_*Hsp20A
*hsp20B*	pET24a(+)_*hsp20B*	*E. coli_*Hsp20B
/	pHT01	*B. th*_WT
*hrcA*	pHT01_ *hrcA*	*B. th*_ HrcA
*hsp20A*	pHT01_ *hsp20A*	*B. th*_ Hsp20A
*hsp20B*	pHT01_ *hsp20B*	*B. th*_ Hsp20B
*fliC*	pHT01_ *fliC*	*B. th*_ FliC
*rpsD*	pHT01_ *rpsD*	*B. th*_ RpsD

a *hrcA*: heat-inducible transcriptional repressor; cot*M*: spore coat protein M; *ctsR*: transcriptional regulator of stress and heat shock response; *hsl*R: ribosome-associated heat shock protein Hsp15; *hsp20A* and *hsp20B*: HSP20 family proteins from *B. thermoamylovorans* (BtHsp20A and BtHsp20B); *fliC*: flagellin; *rpsD*:30S ribosomal protein S4 from *B. thermoamylovorans*.

b Constructed plasmid containing genes (plasmid_gene).

c Strains with pET24a(+)/pHT01 containing thermotolerant devices; *B. th* (*Bacillus thermoamylovorans*); *E. coli* (*E. coli* BL21 (DE3)); *E. coli*_WT/*B. th*_WT (wild-type strains with plasmid of pET24a(+)/pHT01).

### 2.5 Effect of thermotolerant gene overexpression on cell heat resistance

Single colonies of the engineered strains were inoculated into 10 mL of LB broth supplemented with 30 µg/mL kanamycin (Kan^R^) for *E. coli* at 37 °C or 12.5 µg/mL Cm^R^ for *B. thermoamylovorans* at 50 °C and incubated overnight at 200 rpm as seed cultures. The cultures were then diluted to an initial OD_600_ of 0.1 in 50 mL of fresh LB broth containing the same antibiotic concentration (0.1 mM isopropyl-β-d-thiogalactopyranosidefor *E. coli*). Subsequently, these cultures were incubated at different temperatures (37, 42, and 45 °C for *E. coli*; 50, 60, and 70 °C for *B. thermoamylovorans*) under the same conditions. During incubation, cell growth was monitored by measuring the OD_600_. After incubation, *B. thermoamylovorans* cells were harvested for spot dilution analysis to evaluate their thermotolerance.

### 2.6 PET degradation analysis using the engineered *B. thermoamylovorans*

The seed cultures of engineered *B. thermoamylovorans* were adjusted to an OD_600_ of 5.0, centrifuged at 4000 rpm for 5 min at 4 °C, and washed 2–3 times with an equal volume of sterile saline (0.95 % NaCl). Cell pellets were subsequently transferred at 1 % (w/v) inoculation into M9 broth containing 12.5 µg/mL Cm^R^ and 100 mg PET as the sole carbon source. During incubation at 50 °C and 200 rpm, cell growth was measured by monitoring OD_600_, and the strain exhibiting the highest cell density was selected for further quantification of TPA released from PET degradation. Finally, residual PET was analyzed using scanning electron microscopy (SEM).

### 2.7 Adaptability of engineered *B. thermoamylovorans* in an oligotrophic ecosystem

The cell pellets of engineered *B. thermoamylovorans* were transferred at 1 % (w/v) inoculation into M9 broth containing 0.2 % peptone, 0.1 % yeast extract, and 12.5 µg/mL of Cm^R^ with either PET (100 mg), TPA (25 mg), or a PET+TPA mixture. During incubation at 50 °C and 200 rpm, cell growth was measured every 24 h, and the cells were subjected to sodium dodecyl sulfate-polyacrylamide gel electrophoresis (SDS-PAGE) analysis. Protein bands were excised and analyzed using a Thermo Scientific Q Exactive Plus mass spectrometer for peptide coverage mapping. Additionally, the effect of target protein overexpression on *B. thermoamylovorans* was also investigated through monitoring the OD_600_ and quantifying TPA production from PET as described in Section 2.6.

### 2.8 Analysis methods

#### 2.8.1 Spot dilution analysis

After incubation, the cell suspension was serially diluted from 10^–1^ to 10^–6^ using sterile saline. A 1.5 µL aliquot from each dilution was transferred onto LB-agar plates using a pipette and incubated at 50 °C overnight. The growth of colonies in each droplet was used to qualitatively evaluate heat resistance of the target strain. Heat resistance was characterized by the presence of colonies in each droplet.

#### 2.8.2 TEM analysis

Cells were collected and subjected to TEM analysis. The transmission specimens were analyzed using an H-7650 TEM, equipped with an energy spectrum analyzer, operating at a voltage of 80.0 kV. Then, the cell suspension was dripped onto a carbon-coated copper grid for observation.

#### 2.8.3 SEM analysis

Residual PET was collected and sequentially washed with a 1 % (w/v) sodium dodecyl sulfate solution, deionized water, and a 75 % ethanol solution. After washing, the PET was air-dried and coated using an osmium (Os) plasma-ion coater. The erosion of the PET surfaces was observed using a SU8100 SEM at an electron beam intensity of 10 kV.

#### 2.8.4 SDS-PAGE analysis

Cell pellets were resuspended in 50 mM Tris–HCl (pH 7.5) buffer containing 20 mg/mL lysozyme and incubated at 37 °C for enzymatic lysis. After high-pressure homogenization, the cell lysate was collected by centrifugation at 8000 rpm for 1 min, and then protein integrity was verified via SDS-PAGE analysis [31].

#### 2.8.5 HPLC analysis

The reaction solution was analyzed via HPLC (Agilent 1100) using an Ultimate XB-C18 column (4.6 × 250 mm, 5 µm, Welch). The mobile phase consisted of methanol (HPLC grade) and 1 % (w/v) acetic acid (35:65, v/v) at a flow rate of 0.5 mL/min. Detection was performed at 240 nm using a UV detector, and monomer yield was calculated against standard curves [30].

### 2.9 Statistical analysis

All statistical analyses were performed with the SPSS 25.0 statistical package (IBM, Armonk, NY, USA). Values are presented as the mean ± standard deviation from three independent experiments. Graphs were prepared in Graphpad Prism (version 7.02).

## 3 Results and discussion

### 3.1 Heat resistance analysis of wild-type *B. thermoamylovorans*

**Fig. 1:**
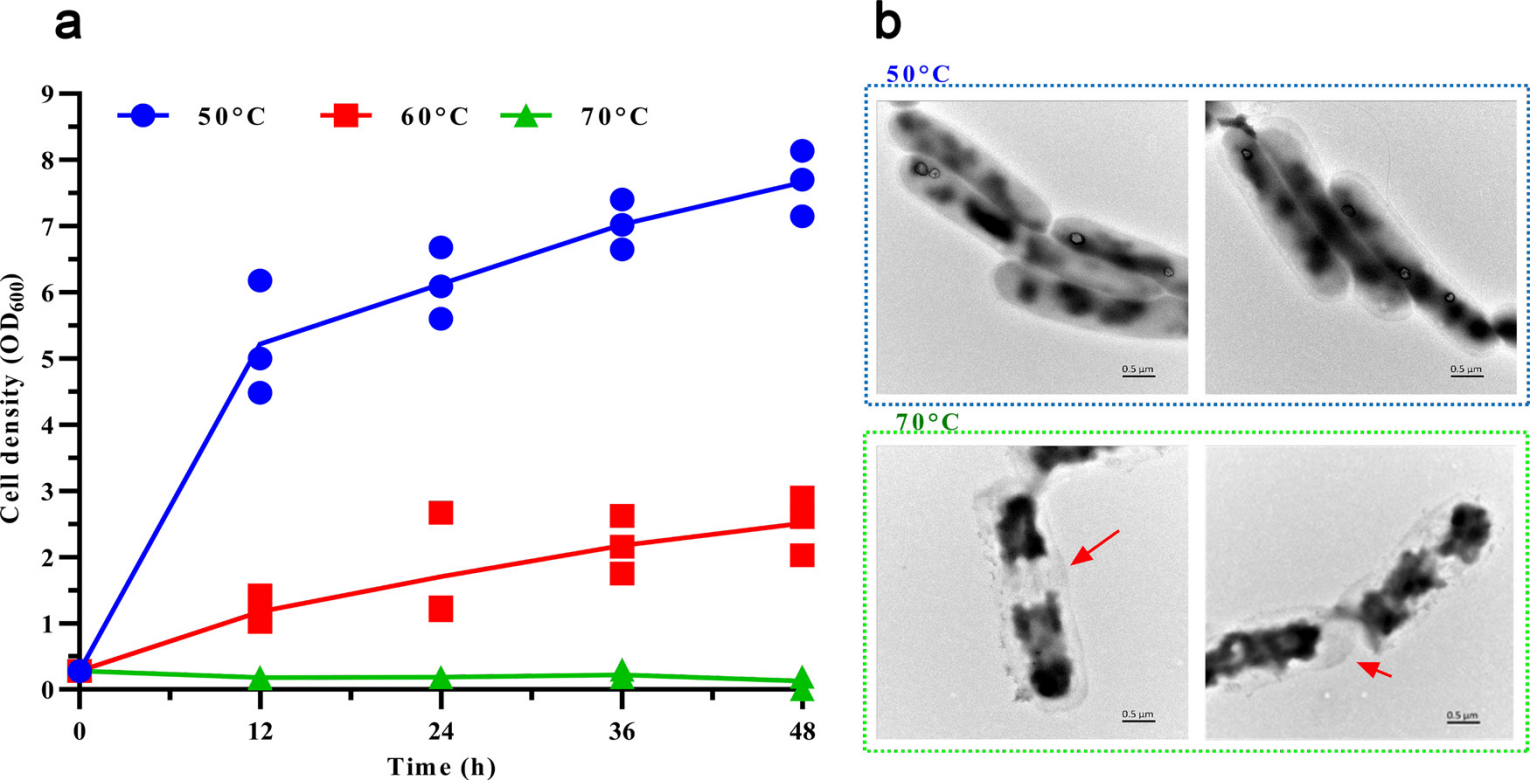
Analysis of heat resistance in *B. thermoamylovorans* at different temperatures. (a) Growth curves of *B. thermoamylovorans* at 50, 60, and 70 °C, respectively. The cell density at 600 nm (OD_600_) was measured every 12 h for 48 h. (b) TEM images of *B. thermoamylovorans* after 48 h of cultivation at 50 and 70 °C, respectively.

Thermophiles have uniquely adapted to thrive in high-temperature environments [20,32]. The growth curves (Fig. 1a) revealed that *B. thermoamylovorans* exhibited optimal growth at 50 °C, with moderated viability at 60 °C and significant inhibition at 70 °C. After 12 h of incubation, the growth rate was 0.41 OD_600_/h at 50 °C but declined to 0.10 OD_600_/h at 60 °C, indicating a 75.6 % loss in cell activity. TEM analysis (Fig. 1b) showed that *B. thermoamylovorans* cells maintained their structural integrity at 50 °C, exhibiting well-defined, rod-like structures and preserved membrane integrity after 48 h of incubation. However, incubation at 70 °C led to cell damage, including vesicle collapse, cytoplasmic leakage, and large gaps in the peptidoglycan layer. These observations are characteristic of thermal injury and ultimately result in cell lysis. The cellular structure of *B. thermoamylovorans* consists of three layers: a relatively thick outermost layer, a thinner middle layer, and an innermost layer known as the cell membrane. Numerous crypts are present between the outer and middle layers, which may enhance thermal stability [33]. The cell membrane had a high concentration of saturated fatty acids, promoting robust hydrophobic interactions and reinforcing the stability of the membrane structure at high temperatures [34]. These findings indicated that high temperatures disrupt cell membrane integrity and cellular functions, leading to inhibited cell division, damage to intracellular organelles, and cell death.

### 3.2 Identification of thermotolerant genes in *B. thermoamylovorans*

**Fig. 2:**
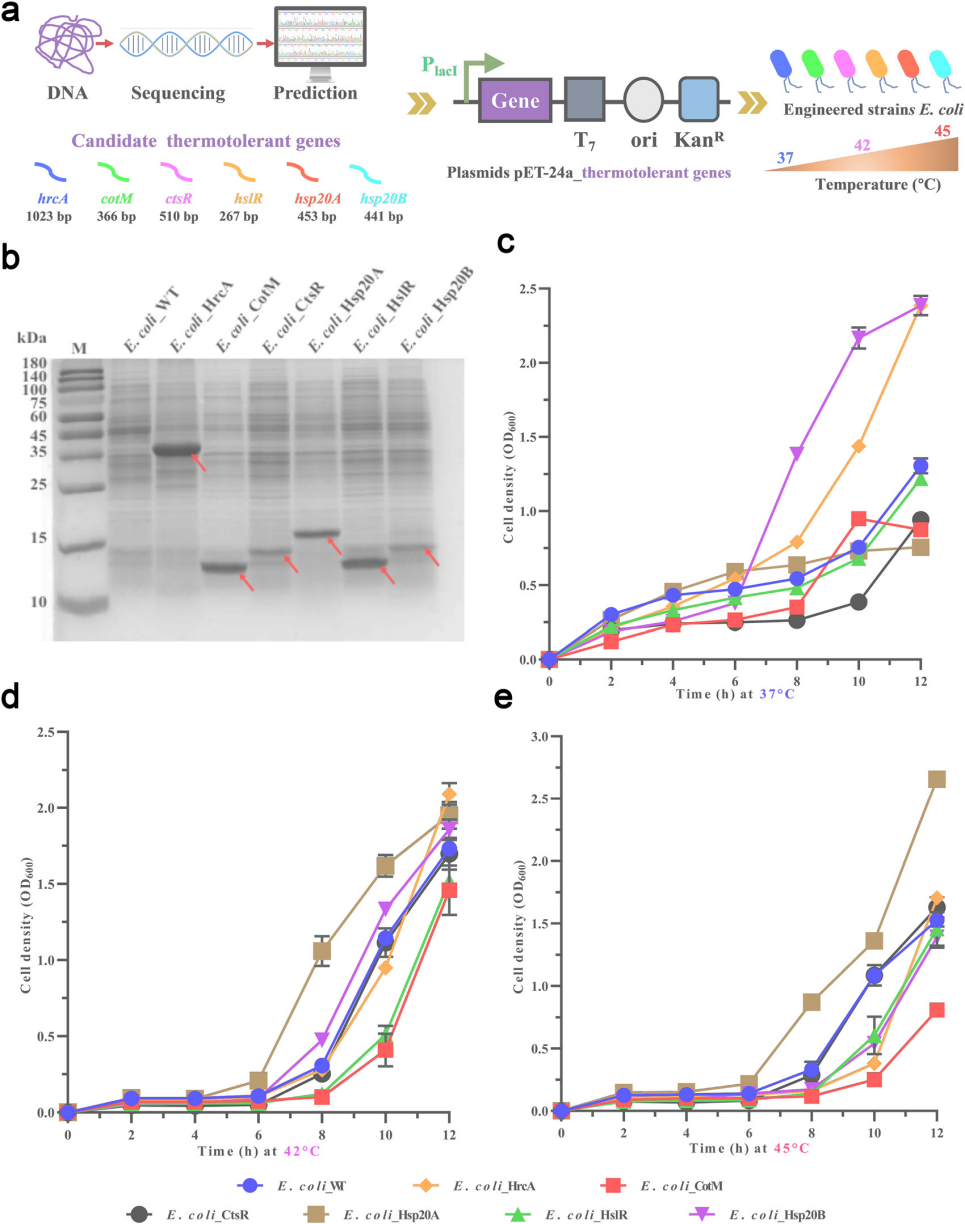
Identification and functional verification of thermotolerant genes from *B. thermoamylovorans* in *E. coli*. (a) Schematic diagram of thermotolerant gene identification and functional verification using a cassette in *E. coli* as the host. (b) SDS-PAGE analysis of cell lysates in engineered *E. coli*. The red arrows represent the proteins expressed by their corresponding thermotolerant genes. (c–e) Growth curves of engineered *E. coli* at 37 °C (c), 42 °C (d), and 45 °C (e).

The high thermotolerance exhibited by thermophiles in their natural habitats is attributed to the acquisition of stress-responsive genes through evolutionary adaptations [20,34]. In this study, six thermotolerant genes were identified within the genome of *B. thermoamylovorans*, including *hrcA*, cot*M, ctsR, hslR, hsp20A,* and *hsp20B* (Fig. 2a). Each of these gene was successfully heterologously expressed in *E. coli* to assess their effects on heat resistance (Fig. 2b). As shown in Fig. 2c, *E. coli_*Hsp20B and *E. coli_*HrcA exhibited higher growth at 37 °C than the wild type *E. coli* (*E. coli*_WT), with *E. coli_*Hsp20B exhibiting the highest growth rate, reaching an OD_600_ of 2.4 after 12 h of incubation. This suggested that the protein encoded by *hsp20B* may support metabolic activity at moderate temperatures. During incubation at 42 °C (moderate heat stress) (Fig. 2d), *E. coli_*Hsp20A showed the highest cell density, followed by that of *E. coli_*Hsp20B, while other genes had no observable effect on *E. coli* growth. These results indicated that *hsp20A* might provide protective effects by stabilizing nascent proteins during translation under moderate-to-high heat stress. These results are consistent with previous findings regarding hyperthermophilic archaeon *Sulfolobus solfataricus*, in which overexpression of the thermotolerant gene *hsp20* in *E. coli* also provided heat protection [32]. At 45 °C (Fig. 2e), *E. coli_*Hsp20A showed an obvious increase in cell density, reaching an OD_600_ of 2.6 after 12 h of incubation. The protein encoded by *hsp20A* is recognized as a high-temperature-specific chaperone, assisting in protein repair or membrane stabilization. This role is similar to that of CeHsp17 from *Caenorhabditis elegans* [25], which also protects against heat stress. Notably, the growth advantage of *E. coli*_HrcA at 37 °C is closely related to the regulatory mechanism of HrcA. As a key transcriptional repressor of the heat shock response, HrcA specifically binds to the CIRCE element in the promoters of major chaperone operons, such as *dnaK* and *groE*, effectively suppressing the expression of these energy-costly proteins [35,36]. This repression enables the cell to reallocate metabolic resources (including ATP, amino acids, and ribosomes) originally dedicated to chaperone synthesis toward biosynthetic processes, such as cell growth and division, thereby conferring a competitive advantage under optimal temperature conditions [37,38]. In contrast, the delayed growth recovery observed under heat stress at 42–45 °C reveals the complexity of the bacterial stress response network. Under *hrcA* overexpression, a higher concentration of misfolded proteins is required to relieve its repression, thereby delaying the initiation of the heat shock response [39]. However, as stress persists, cells may activate alternative stress pathways, such as those mediated by σ⁵⁵ [40], or undergo global metabolic remodeling, thereby gradually establishing *hrcA*-independent compensatory mechanisms that ultimately restore growth [41]. In this study, a time-dependent protective effect was also evident in *E. coli* overexpressing these thermotolerant genes. Growth differences between engineered strains were minimal in the first 8 h, indicating delayed activation of the stress response. However, the growth rates diverged significantly between 8 and 12 h (Fig. S2), which indicated the phase-specific role of these genes in exponential growth and heat adaptation. These results suggested that the thermotolerant genes function at specific stress levels and time windows, forming a coordinated defense system.

### 3.3 Heat resistance analysis of *B. thermoamylovorans* overexpressing thermotolerant genes

**Fig. 3:**
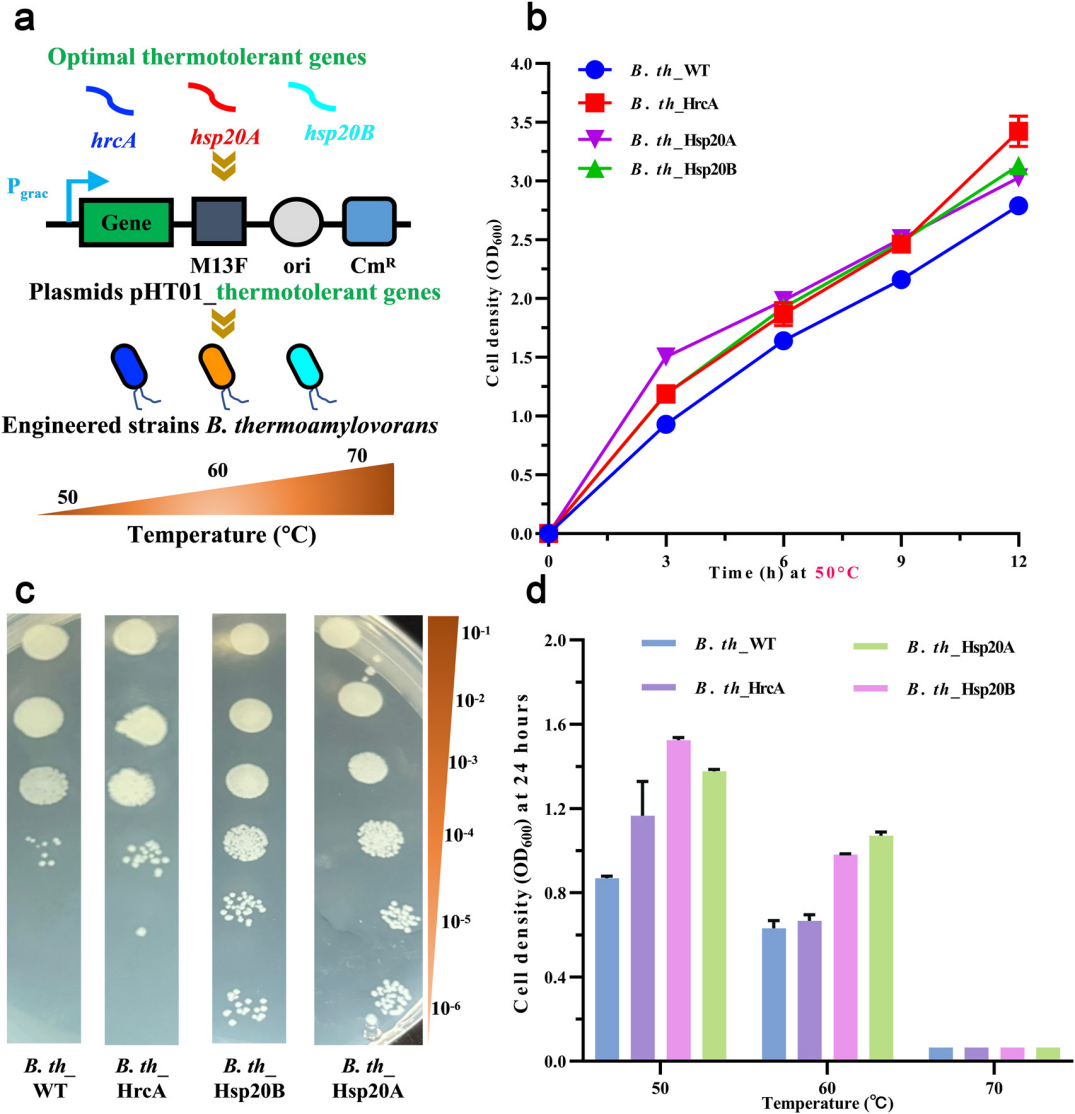
Heat resistance analysis of *B. thermoamylovorans* with thermotolerant gene overexpression. (a) Schematic diagram of the thermotolerant gene expression cassette in *B. thermoamylovorans* and its functional verification. (b) Growth curve of engineered strains in Luria-Bertani broth at 50 °C. (c) Spot dilution analysis of engineered strains. (d) Cell density of engineered strains in LB broth after 24 h of incubation at 50, 60, and 70 °C.

Based on the results observed in *E. coli*, three potential thermotolerant genes (*hrcA, hsp20A,* and *hsp20B*) were selected for overexpression in *B. thermoamylovorans* to evaluate their impact on heat resistance (Fig. 3a). During incubation at 50 °C, the OD_600_ values of all engineered *B. thermoamylovorans* were significantly higher than those of the wild type (*B. th*_WT), indicating that overexpression of thermotolerant genes enhanced cell growth at 50 °C (Fig. 3b). This finding is consistent with those of Chen et al., who reported that overexpression of the gene *TrRCC1* from *Trichoderma reesei* in *S. cerevisiae* significantly enhanced thermotolerance and increased ethanol production by 21.8 % [42]. Furthermore, spot dilution assays revealed that the engineered strains exhibited greater thermal resilience than *B. th*_WT. Among them, *B. th_*Hsp20A showed the highest colony-forming ability, followed by *B. th_*Hsp20B and *B. th_*HrcA (Fig. 3c). As shown in Fig. 3d, the engineered strains of *B. thermoamylovorans* grew optimally at 50 °C, with moderate growth at 60 °C. Notably, *B. th_*Hsp20B achieved the highest cell density, with an OD_600_ of 1.52 at 50 °C, which was 1.8 times higher than that of *B. th_*WT. However, *B. th_*Hsp20A displayed the highest growth (OD_600_ =1.07) at 60 °C, followed by *B. th_*Hsp20B (0.98). In contrast, *B. th_*HrcA showed no significant advantage over *B. th_*WT. None of the strains were able to grow at 70 °C, indicating that this temperature exceeds the thermal tolerance of *B. thermoamylovorans*, even with HSP-assisted protection. These observations are consistent with previous findings of studies on *E. coli* (Fig. 2), supporting that overexpression of thermotolerant genes, particularly *hsp20A*, can improve thermotolerance, colony viability, and the overall heat stress tolerance of *B. thermoamylovorans*.

### 3.4 Analysis of PET degradation by the engineered strain *B. th_*Hsp20A

**Fig. 4:**
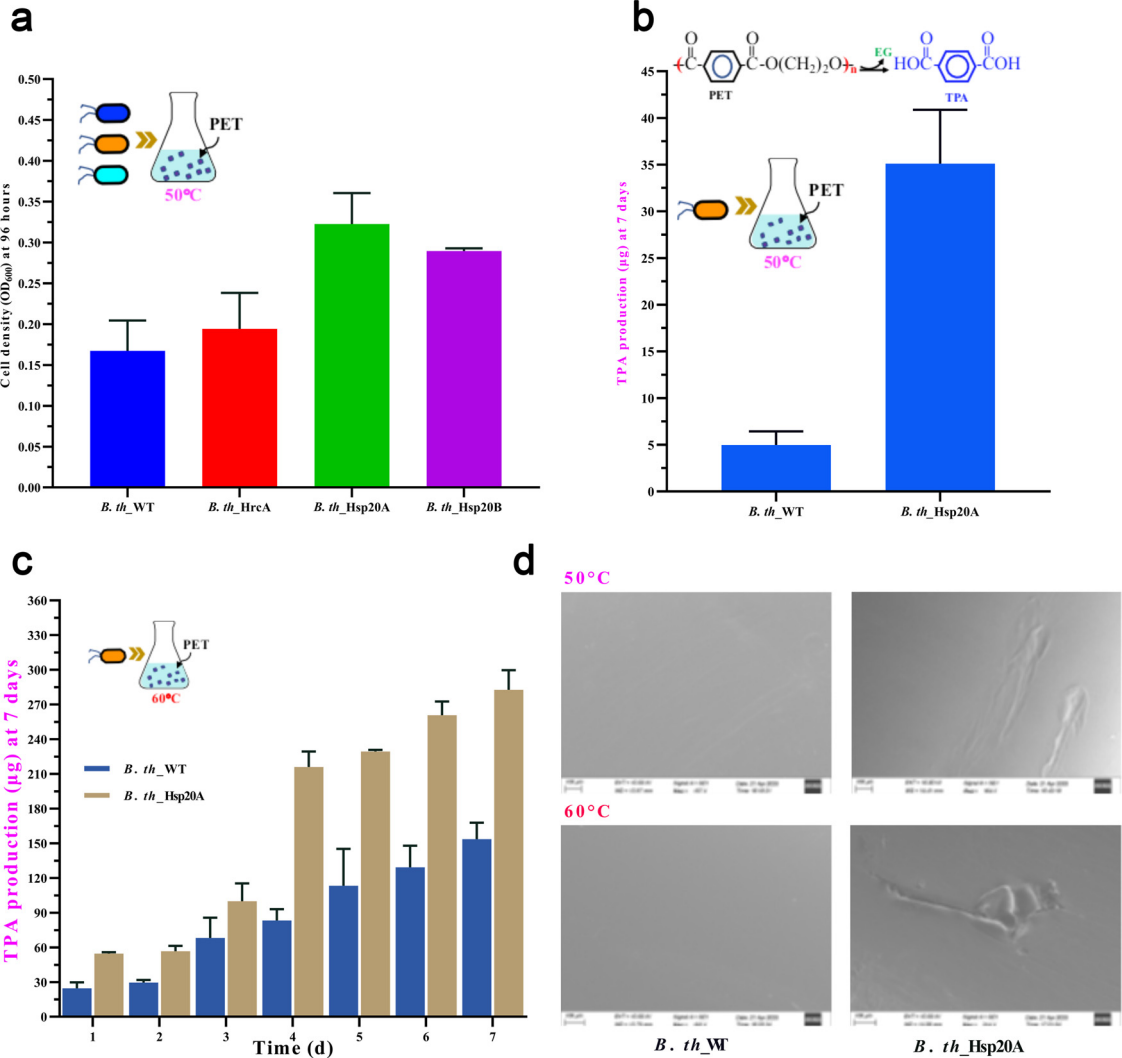
PET degradation analysis of the engineered strain *B. th_*Hsp20A. (a) Cell density of the engineered strains after 96 h of incubation with 100 mg PET at 50 °C. (b) TPA production by the engineered strains after 7 days of incubation with 100 mg of PET at 50 °C. (c) TPA release from PET degradation by *B. th_*Hsp20A at 60 °C. (d) SEM images of the PET surface after degradation.

As shown in Fig. 4a, *B. th_*Hsp20A exhibited the highest utilization rate of PET, with an OD_600_ of 0.38 after 96 h of incubation at 50 °C, followed by those of *B. th_*Hsp20B (0.28), *B. th_*HrcA (0.21), and *B. th_*WT (0.16). The TPA production quantification results (Fig. 4b) also indicated that *B. th_*Hsp20A released a higher level of TPA (35.1 µg) from PET after 7 days of incubation at 50 °C, which was 7.6 times higher than that of *B. th_*WT (5.2 µg). Moreover, *B. th_* Hsp20A accumulated a higher level of TPA at 60 °C compared to 50 °C, reaching 282 µg after 7 days of incubation, which was double the amount released by *B. th_*WT (Fig. 4c). SEM analysis (Fig. 4d) revealed more severe surface erosion of PET by *B. th_*Hsp20A, indicating that overexpression of *hsp20A* improves thermal tolerance, survival in nutrient-limited environments, and PET utilization. This enhanced performance is likely due to the synergistic effects of thermotolerant and improved metabolic activity, which enables *B. th_*Hsp20A to better utilize PET under high temperatures.

### 3.5 Structure-activity relationship analysis of BtHsp20A

**Fig. 5:**
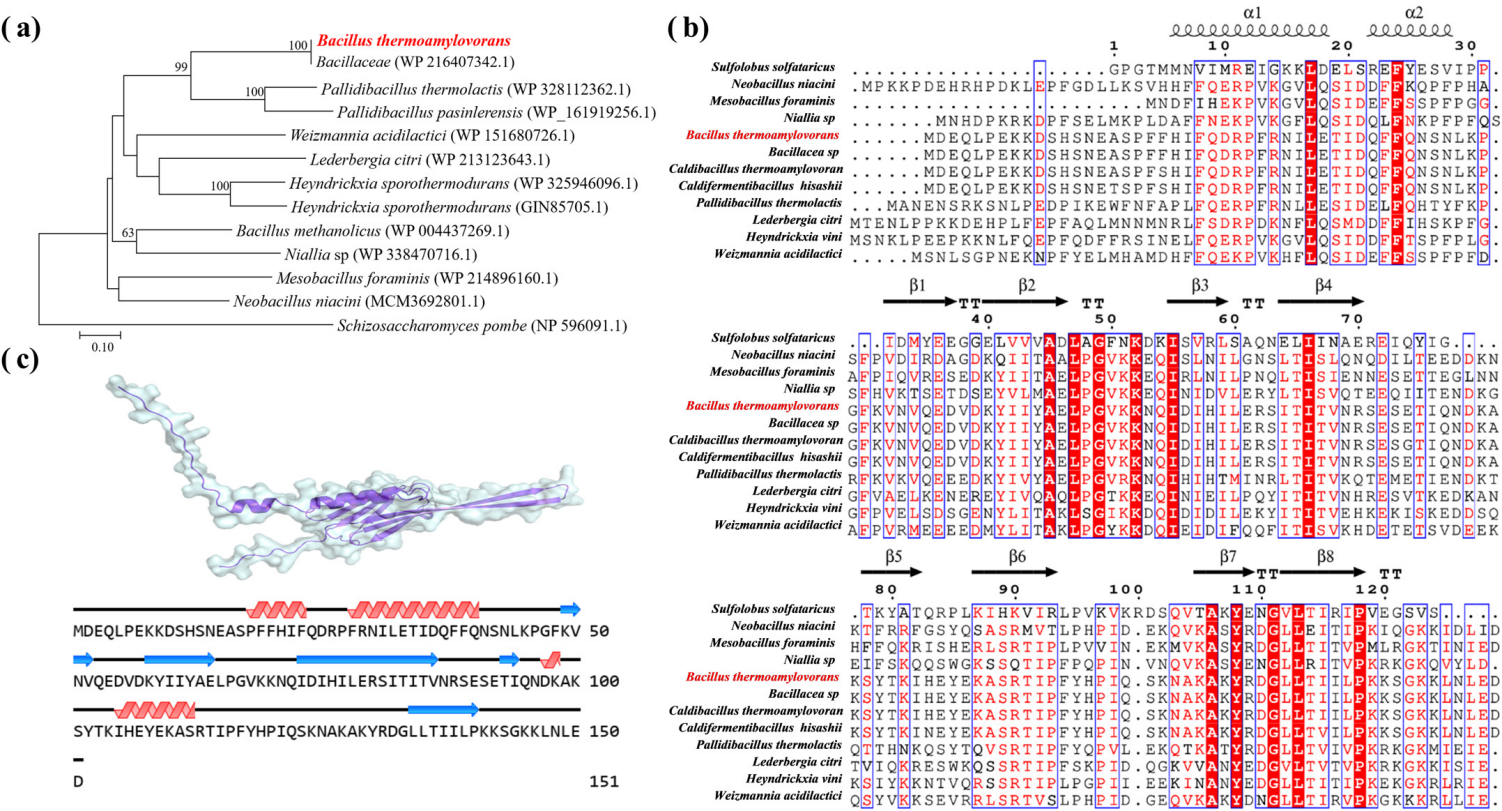
Sequence analysis and structure prediction of BtHsp20A. (a) Maximum likelihood tree of BtHsp20A and HSPs from other thermophiles based on amino acid sequences. Numbers at nodes are bootstrap values (%) based on 2000 replicates. Values < 50 % are not shown. BtHsp20A is indicated by a red-colored star. (b) Multiple sequence comparison of BtHsp20A with HSPs from other thermophiles. (c) Structure prediction of BtHsp20A using AlphaFold3 and Novopro.

Phylogenetic analysis and structure prediction of the protein BtHsp20A, encoded by the gene *hsp20A* were conducted, and the results are shown in Fig. 5. BtHsp20A was classified as a member of the Hsp20/α-crystallin protein family, which was characterized as a small heat shock protein (sHSP) with an approximate molecular weight of 20 kDa. HSPs are a superfamily conserved across all domains of life, including archaea, bacteria, and eukaryotes [43]. sHSPs, as molecular chaperones, can bind to denatured proteins during environmental stress, thereby preventing irreversible protein aggregation [28,44,45]. This chaperone activity is critical for microbial thermotolerance, as overexpression of HSP genes enhances cellular defense against diverse stressors [45,46]. Phylogenetic analysis (Fig. 5a–b) showed that BtHsp20A clustered closely with thermophiles, such as *Heyndrickxia sporothermodurans, Pallidibacillus thermolactis*, and *Bacillus methanolicus*, indicating evolutionary selection for heat resilience. Secondary-structure prediction revealed that BtHsp20A is composed of 151 amino acids, with 31.8 % α-helices, 19.9 % β-sheets, and 48.3 % flexible coils. The high proportion of disordered regions indicated that BtHsp20A functions as a “holdase” chaperone, which enables versatile interactions with misfolded substrates under stress conditions [16,21]. Structural modeling (Fig. 5c) revealed that BtHsp20A showed a compact β-sandwich α-crystallin domain. The protein is flanked by an N-terminal helix, which is involved in oligomer nucleation, and a C-terminal β-strand arm rich in hydrophobic residues, such as Ile, Val, and Leu. The N-terminal α-helix region (α1–α2) is likely involved in protein oligomerization and initial substrate recognition. The C-terminal β-sheet-rich domain (β4–β8) is highly conserved, which contributes to the stabilization of substrate proteins and oligomer formation. Several conserved hydrophobic residues (e.g., I/V/L, highlighted in red in Fig. 5b) within the β-sheet regions are critical for substrate interaction and thermal stabilization, which is consistent with the known functional mechanisms of HSPs [45,46].

### 3.6 Adaptability of engineered *B. th_*Hsp20A in an oligotrophic ecosystem

**Fig. 6:**
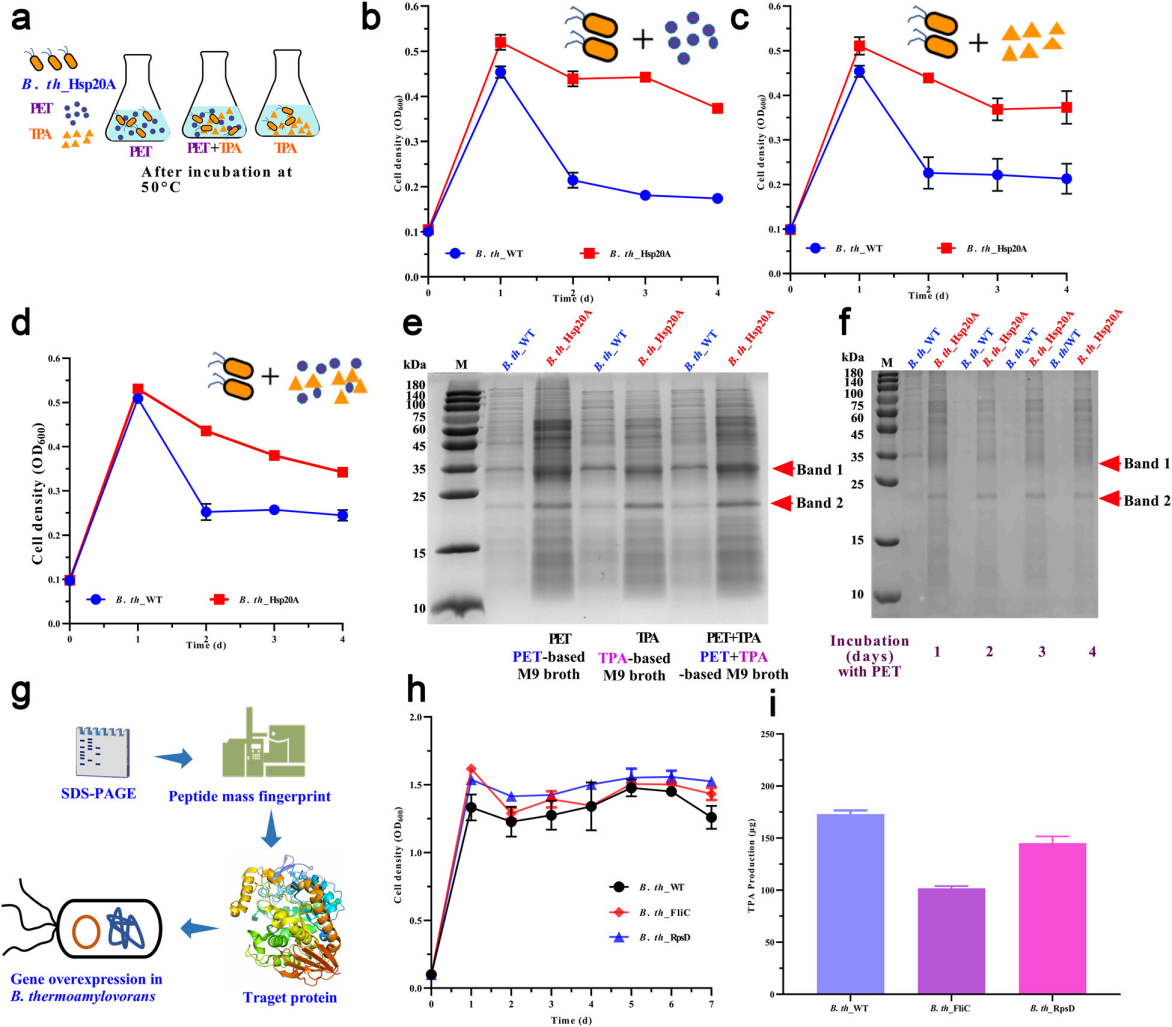
Adaptability analysis of engineered *B. th*_Hsp20A in an oligotrophic ecosystem. (a) Schematic diagram of adaptability analysis. Growth curve of *B. th_*Hsp20A in M9 broth containing PET (b), TPA (c), and PET plus TPA (d) at 50 °C. (e) SDS-PAGE analysis of cell lysates after 1 day of incubation. (f) SDS-PAGE analysis of cell lysate over time in PET-based M9 broth. (g) Schematic diagram of target protein identification and verification. Cell density (h) and TPA production (i) of the engineered strain *B. thermoamylovorans*.

In this study, PET, TPA, and a PET+TPA mixture were designed as oligotrophic ecosystems to evaluate the adaptability of *B. th*_Hsp20A (Fig. 6a). As shown in Fig. 6b–d, both *B. th*_Hsp20A and *B. th_*WT reached peak OD_600_ after 1 day of incubation at 50 °C, which subsequently declined. Compared to *B. th_*WT, *B. th*_Hsp20A exhibited significantly higher cell density and a more gradual decline. This decline is common in response to heat stress, often accompanied by the denaturation and degradation of intracellular proteins [28]. HSPs can inhibit the activation of protein kinases and the transduction of apoptosis signals, thereby protecting cells from premature death [43,47]. As shown in Fig. 6e, the cell lysate of *B. th_*Hsp20A maintained higher levels of protein synthesis in oligotrophic ecosystems compared to *B. th_*WT, suggesting that BtHsp20A might extend cellular metabolic activity during heat stress by stabilizing key enzymes and preventing proteolytic degradation. During incubation in PET-based M9 broth (Fig. 6f), *B. th_*Hsp20A demonstrated sustained synthesis of soluble proteins, indicating its role in prolonging the metabolic activity window by maintaining proteostasis. As a member of the α-crystallin sHSP family, BtHsp20A contains a hydrophobic β-sheet core, which can encapsulate hydrophobic segments (e.g., PETases, MHETases, or membrane transporters) under heat stress, preventing their inactivation. Additionally, sHSPs can “hold” damaged proteins in the absence of ATP, allowing other chaperone proteins to refold them more efficiently [25,[44], [45], [46]]. This feature is consistent with the dual thermal and nutrient stress encountered during PET biodegradation.

Two protein bands with high abundance were excised from the SDS-PAGE gel (Fig. 6f) and subjected to mass spectrometer analysis (Fig. 6*g*). These two bands were identified as flagellin (Band 1) and 30S ribosomal protein S4 (Band 2). The findings from the analysis are presented in Table S3 and Fig. S3, while comprehensive datasets are provided in Supplementary Table S4–S7. Based on the amino acid sequences, the genes encoding *filC* for flagellin and *rpsD* for ribosomal protein were identified and amplified from the genomic DNA of *B. thermoamylovorans*. The effect of overexpressing *filC* and *rpsD* in *B. thermoamylovorans* on heat resistance was investigated. As shown in Fig. 6h, both *B. th*_FilC and *B. th*_RpsD showed no significant difference in cell density compared to *B. th*_WT at 60 °C. However, their TPA production was lower than that of *B. th*_WT (Fig. 6i), particularly for *B. th*_FilC. These results indicated that although overexpression of BtHsp20A enhances the synthesis of both flagellin and ribosomal proteins in *B. thermoamylovorans*, these proteins are not the primary factors responsible for the improved PET degradation capability. In *B. th*_Hsp20A, the enhanced biofilm formation associated with flagellin overexpression might provide a more stable microenvironment, which could explain the slightly higher cell density of *B. th*_FilC compared to *B. th*_WT after 1 day of incubation. Meanwhile, ribosomal protein is crucial for stabilizing the ribosomal subunit and ensuring continued protein synthesis at high temperatures [48,49]. Both proteins are important for maintaining metabolic activity and protein stability in *B. thermoamylovorans* under heat stress, which is essential for sustaining both growth and PET degradation at high temperatures.

## 4 Conclusion

In this study, six endogenous thermotolerant genes from *B. thermoamylovorans* were identified, and their overexpression in both *E. coli* and *B. thermoamylovorans* significantly enhanced cell density under heat stress. Notably, overexpression of *hsp20A* in *B. thermoamylovorans* (*B. th*_Hsp20A) resulted in an 84.3 % increase in PET degradation at 60 °C compared with the wild type strain, highlighting the positive effect of its overexpression on microbial performance under heat stress. The protein BtHsp20A encoded by *hsp20A* contains a compact β-sandwich α-crystallin domain, which is critical to its chaperone activity. Moreover, overexpression of *hsp20A* demonstrated a synergistic effect on metabolic activity, enhancing survival and protein stability in nutrient-limited environments, thereby facilitating more efficient PET utilization. This work proposes a novel strategy for enhancing microbial PET degradation through engineering thermotolerance. However, further investigations are needed to fully understand the molecular mechanisms underlying the relationship between thermotolerance and PET degradation in *B. thermoamylovorans*.

## Data availability statement

The data will be made available upon request.

## CRediT authorship contribution statement

**Yi-Mei Cai:** Writing – original draft, Methodology, Investigation, Formal analysis. **Kang-Qi Hu:** Methodology, Investigation, Formal analysis. **Xin-Yu Zhang:** Investigation, Formal analysis. **Hui Liu:** Investigation, Formal analysis. **Yan Huang:** Investigation, Formal analysis. **Wei Xia:** Investigation, Formal analysis. **Jing Wu:** Conceptualization. **Zheng-Fei Yan:** Writing – review & editing, Supervision, Project administration, Funding acquisition.

## Declaration of competing interest

The authors declare that they have no known competing financial interests or personal relationships that could have appeared to influence the work reported in this paper.

## Appendix Supplementary materials

Image, application 1

Image, application 2

Image, application 3

Image, application 4

Image, application 5

Image, application 6

Image, application 7
