# Supplementary material for: Enhancement of heat resistance of Bacillus thermoamylovorans drives enhanced PET degradation
Source: Eng Microbiol. 2025 Dec 23;6(2):100256. doi: 10.1016/j.engmic.2025.100256 (PMC13241884; doi:10.1016/j.engmic.2025.100256)
Supplement: Supplementary file 1 [file mmc1.docx]

# Supplementary information

# Enhancement of heat resistance of *Bacillus thermoamylovorans* drives enhanced PET degradation

**
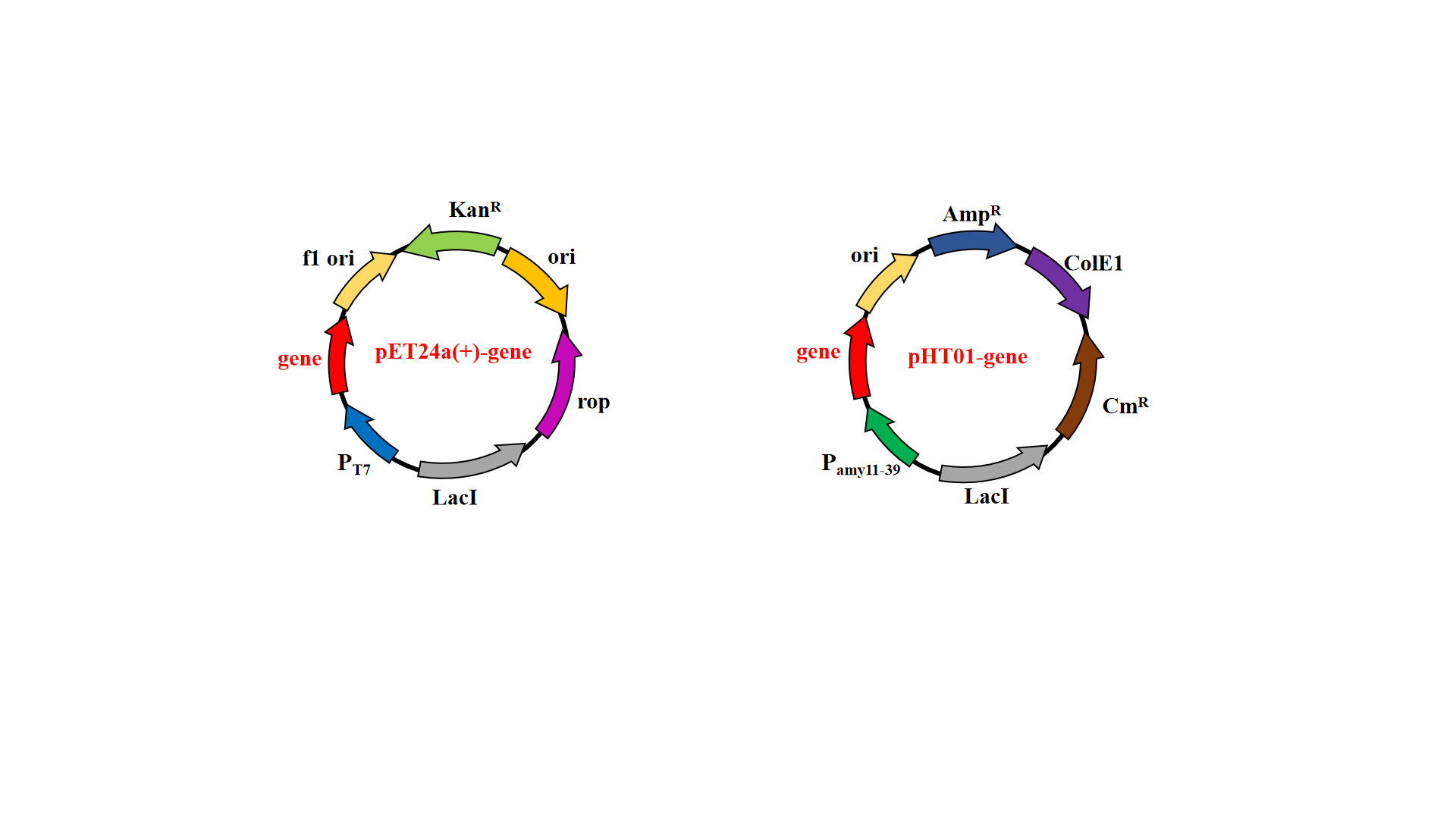
**

**Supplementary Figure S1. Plasmids used for the thermotolerance devices. The two plasmids**

**pET24a(+)_gene/pHT01_gene thermotolerance system.**

**
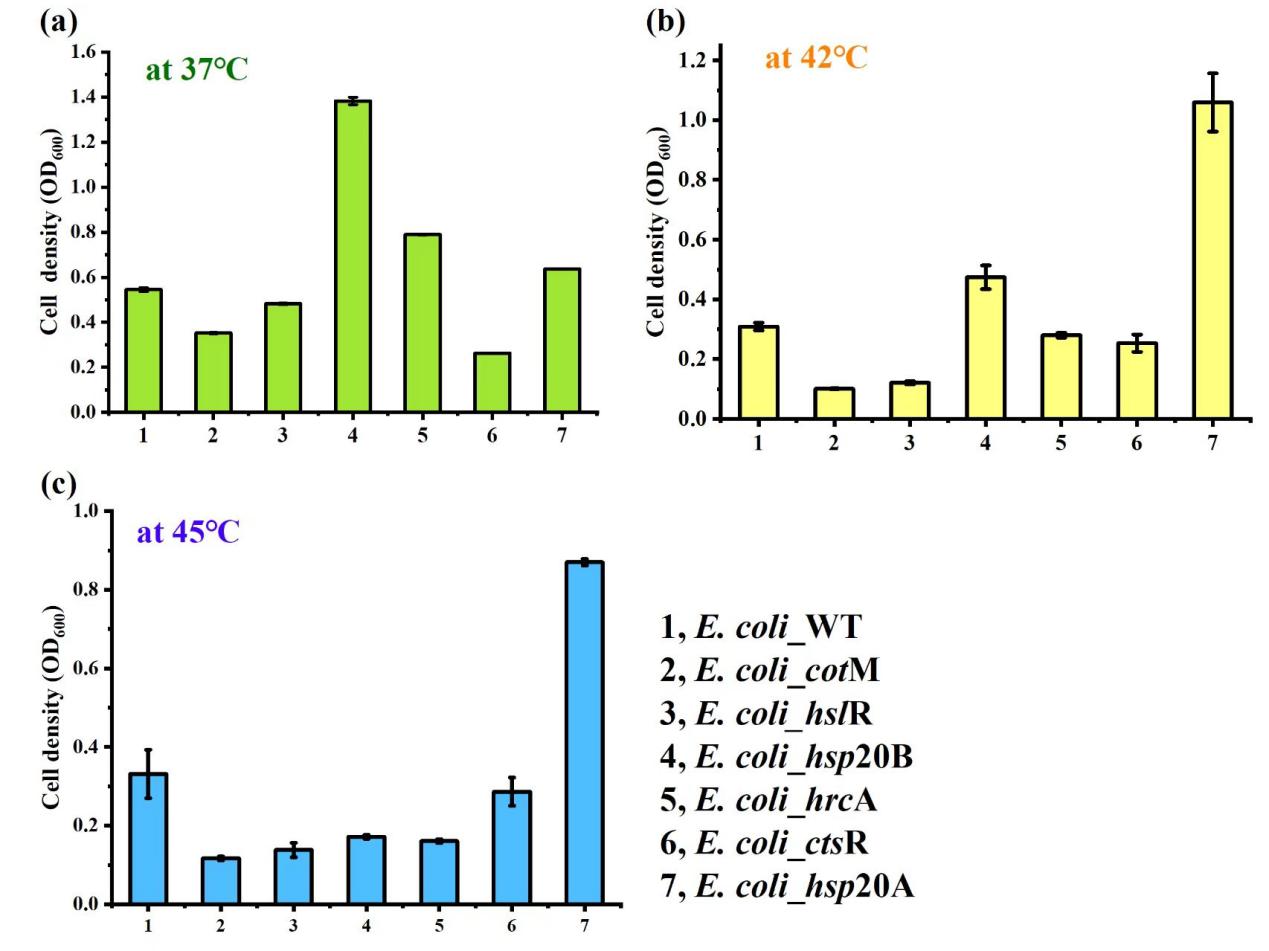
**

**Supplementary Figure S2. Cell density (OD_600_) of engineered *E. coli.* in LB broth at 37 °C (a), 42°C (b), and 45°C (c) for 8 hours, respectively.**

**
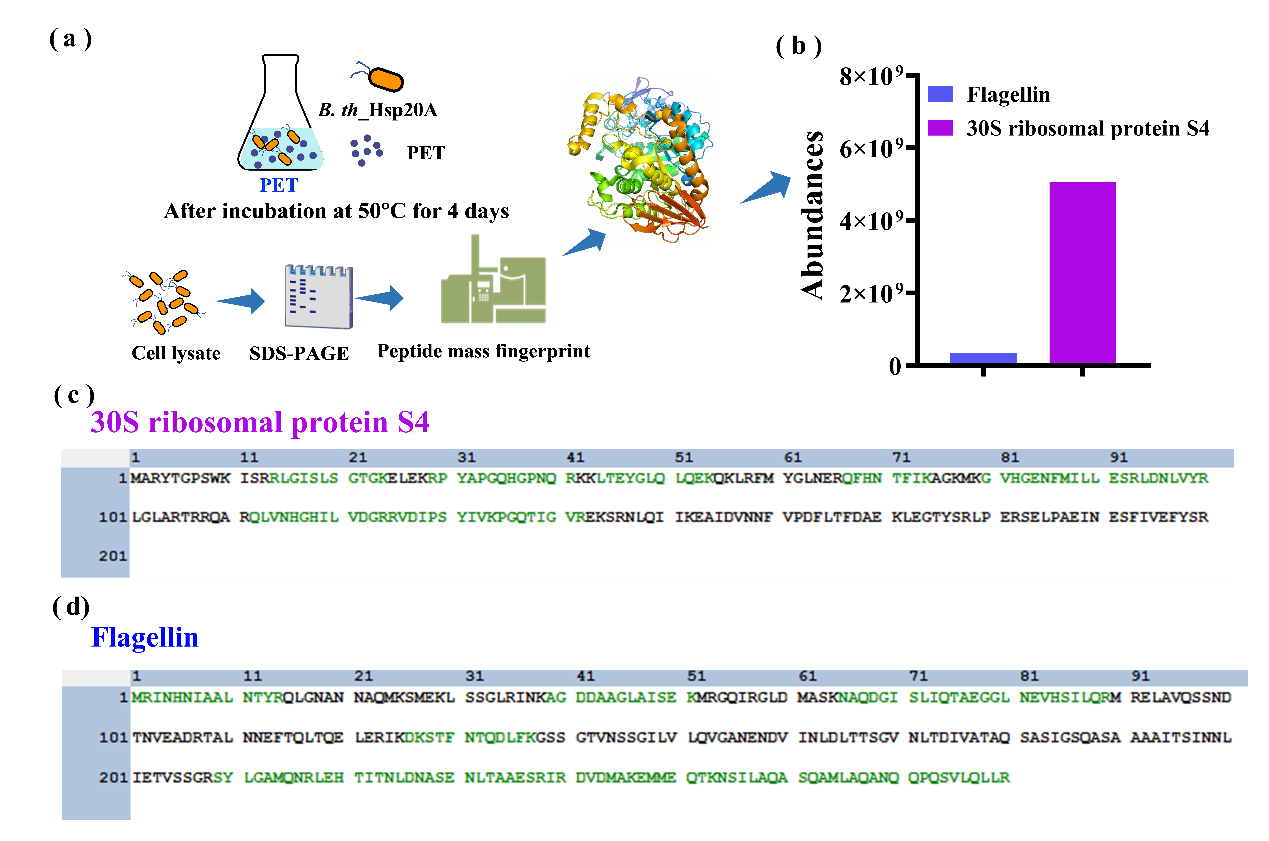
**

**Supplementary Figure S3. (a), Schematic representation of bacterial cultivation and mass spectrometry analysis for *B.th*_Hsp20A; (b), Abundance analysis of 30S ribosomal protein S4 and flagellin; Peptide coverage maps of (c) 30S ribosomal protein S4 and (d) flagellin. 50°C. Cell density (e), and TPA production (f), of engineered *B. thermoamylovorans* in LB broth with 100 mg PET after incubation of 7 days at 60℃.**

**Table S1** Primers information

| Primers | Sequence (5’-3’) |
| --- | --- |
| pHT01_*fliC*-F | AGAAATAAAGGGGGAGAATGATGCGTATTAATCACAATATCGCAGCAC |
| pHT01_*fliC*-R | CCGGGGACGTCGACTCTAGATTATCGTAGTAACTGTAAAACACTTTGAGGCTG |
| pHT01_*rpsD*-F | AGAAATAAAGGGGGAGAATGATGGCTCGTTATACAGGTCCAAGC |
| pHT01_*rpsD*-R | CCGGGGACGTCGACTCTAGATTAACGTGAATAGAACTCAACGATGAACGAT |
| pHT01_*hrcA*-F | AGAAATAAAGGGGGAGAATGTTAACAGAGCGTCAATTGTTAATATTGCAAATCATAATTG |
| pHT01_*hrcA*-R | GGGACGTCGACTCTAGATTATCAGAGATCATATAGCTTTGTTAGTGTAACGGTC |
| pHT01*_cotM*-F | AGAAATAAAGGGGGAGAATGAATGGGAAAGATGAAAACGCCCC |
| pHT01*_cotM*-R | GGGACGTCGACTCTAGATTATTATTTATCTTTTTTGTATAATTTTATTTCAAGTATATCATGTTC |
| pHT01_*ctsR*-F | AGAAATAAAGGGGGAGAATGTTGAGAAATATTTCGGATATCATTGAGGAATATTTAAAGCAAATATTAG |
| pHT01_*ctsR*-R | GGGACGTCGACTCTAGATTATTATTCATACTTTAGAGTTAACAACATTGCTTTTAAAATTCTTGCC |
| pHT01_*hsp20A*-F | AGAAATAAAGGGGGAGAATGGATGAACAATTACCGGAAAAAAAGGACAGC |
| pHT01_*hsp20A*-R | GGGACGTCGACTCTAGATTATCAATCTTCTAAATTCAGTTTCTTCCCGGATTTTTTTG |
| pHT01_*hslR*-F | AGAAATAAAGGGGGAGAATGAGGTTAGATAAATTTTTAAAAGTATCCCGATTGATAAAAAGGC |
| pHT01_*hslR*-R | GGGACGTCGACTCTAGATTATTAGTTTTCTTGATTTATCCGCTCATCTTTCATAACG |
| pHT01_*hsp20B*-F | AGAAATAAAGGGGGAGAATGGCATTAATACCTTATGATCCATTTAGACAATTAACTAACATTAGG |
| pHT01_*hsp20B*-R | GGGACGTCGACTCTAGATTATTAATGGAAATCCACATCAATTCTCTTTTTATTTGTTTGAG |
| pET-24a(+)_*hrc*A-F | AAGAAGGAGATATACATATGTTAACAGAGCGTCAATTGTTAATATTGCAAATCATAATTG |
| pET-24a(+)_*hrc*A-R | CGAGTGCGGCCGCAAGCTTATCAGAGATCATATAGCTTTGTTAGTGTAACGGTC |
| pET-24a(+)*_cotM*-F | AAGAAGGAGATATACATATGATGAATGGGAAAGATGAAAACGCCCC |
| pET-24a(+)*_cotM*-R | CGAGTGCGGCCGCAAGCTTATTATTTATCTTTTTTGTATAATTTTATTTCAAGTATATCATGTTCAAACC |
| pET-24a(+)_*ctsR*-F | AAGAAGGAGATATACATATGTTGAGAAATATTTCGGATATCATTGAGGAATATTTAAAGC |
| pET-24a(+)_*ctsR*-R | CGAGTGCGGCCGCAAGCTTATTATTCATACTTTAGAGTTAACAACATTGCTTTTAAAATTCTTGCC |
| pET-24a(+)_*hsp20A*-F | AAGAAGGAGATATACATATGGATGAACAATTACCGGAAAAAAAGGACAGC |
| pET-24a(+)_*hsp20A*-R | CGAGTGCGGCCGCAAGCTTATCAATCTTCTAAATTCAGTTTCTTCCCGG |
| pET-24a(+)_*hslR*-F | AAGAAGGAGATATACATATGATGAGGTTAGATAAATTTTTAAAAGTATCCCGATTGATAAAAAGG |
| pET-24a(+)_*hslR*-R | CGAGTGCGGCCGCAAGCTTATTAGTTTTCTTGATTTATCCGCTCATCTTTCATAACG |
| pET-24a(+)_*hsp20B*-F | AAGAAGGAGATATACATATGATGGCATTAATACCTTATGATCCATTTAGACAATTAACTAAC |
| pET-24a(+)_*hsp20B*-R | CGAGTGCGGCCGCAAGCTTATTAATGGAAATCCACATCAATTCTCTTTTTATTTGTTTGAG |

**Table S2** All sequences of genes

|  | Sequence (5’-3’) |
| --- | --- |
| *fliC* | ATGCGTATTAATCACAATATCGCAGCACTGAACACATATCGCCAATTAAATGCTGCAAATACTGCACAATCAAAATCAATGGAAAAATTATCTTCTGGTCTTCGTATTAATAAAGCAGGAGATGATGCGGCTGGTCTAGCAATTTCTGAAAAAATGCGGGGCCAAATTCGTGGTTTAGACATGGCTTCTAAAAATGCCCAAGATGGGATTTCATTAATCCAAACAGCAGAAGGTGGACTAAATGAAGTACATTCCATTCTGCAACGTATGAGAGAATTAGCTGTTCAATCTTCTAACGATACTAACGTTGTTGCTGATAGAACGGCACTAAATGATGAGTTTACACAATTAACTAAAGAATTAACGAGAATTAAAGACAAATCAACGTTTAATACTCAAGATTTGTTCAAAGGTGGAGCAGGCACTGCTAACTCTTCCGGTAAGTTAGTCTTACAAGTAGGAGCAAATCAAGATGATACTATTGAACTGGAACTAACAACTTCTGGAGTTGATTTAACAGGTATTGTTTCTTCTGCAACAGCAGCATCAATCGGTTCTCAAGCAAGTGCAGCAGCTGCAATCACTACCATTAACGAACTAATTGAAACTGTATCTTCTGGACGTTCTTATCTAGGAGCTATGCAAAATCGTTTAGAGCATACGATTACAAATCTTGACAATGCGTCTGAAAACTTAACTGCAGCAGAATCTCGTATTAGAGACGTCGATATGGCGAAGGAAATGATGGAGCAAACGAAGAATTCTATTCTTGCTCAAGCTTCTCAAGCAATGCTCGCTCAAGCCAATCAACAGCCTCAAAGTGTTTTACAGTTACTACGATAA |
| *rpsD* | ATGGCTCGTTATACAGGTCCAAGCTGGAAAATTTCCCGTCGTCTTGGTATTTCTTTAAGCGGAACCGGTAAAGAATTAGAAAAACGTCCATATGCCCCGGGGCAACATGGTCCAAACCAACGAAAAAAATTAACTGAATATGGTTTGCAATTACAAGAAAAACAAAAATTACGTTTTATGTACGGCTTGAATGAACGTCAATTCCATAACACATTCATTAAAGCAGGTAAAATGAAAGGTGTTCATGGTGAAAACTTCATGATTTTACTTGAATCTCGCCTTGACAACCTTGTTTACCGCTTAGGATTGGCTCGTACTCGCCGTCAAGCACGCCAATTGGTTAACCATGGTCACATTCTTGTTGATGGTCGTCGCGTTGACATCCCATCTTACATTGTAAAACCGGGTCAAACTATCGGTGTCCGTGAAAAATCACGCAATTTGCAAATCATCAAAGAAGCTATTGATGTGAACAATTTCGTTCCTGACTTCTTAACTTTCGATGCGGAAAAATTAGAAGGTACTTACAGCCGCTTACCGGAACGCTCTGAACTTCCAGCAGAAATAAACGAATCGTTCATCGTTGAGTTCTATTCACGTTAA |
| *hrcA* | TTAACAGAGCGTCAATTGTTAATATTGCAAATCATAATTGATGACTTTATTCGTTCTGCACATCCGGTTGGGTCAAGGAGTTTAGCGAAAAAGAAAGGCATTTCTTTCAGTTCGGCAACCATTCGTAATGAAATGGCAGACTTGGAAGAATTAGGTTTTATTGAAAAAACCCATACATCTTCCGGGCGGGTCCCTTCCGAAAAAGGATATCGCTTTTATGTCGATCATTTGCTTTCCCCTGAAAAGGTAAGTCATAAAGAGATTAAAAGGATTAATGACCTTTTTACGGAAATTTATGAAATGGAAAAAGTTATCCAAAATGCCGCGAAAGTTTTATCGGAATTAACGGAGTATACAGCGATCGTCTTAGGTCCAACAGTAGAGGATAATAAATTAAACAGATTTCAAATTATCCCGCTTAACGCTGAGACTGCCATTGCCGTTATTGTTACAAATAAAGGGCATTTAGAACACAAACTGTTTTCTTTGCCCAAATCGGTTAATTCTTCTGACCTTGAAAAAACCGTAAATATATTGAATGAACGACTTGTAGGAATGCCAATTCTTGAATTGCAATCAAAGTTAATAAATGAAGTTGTCACTTTAATTAAAGAAAATGTCCATAATTATGAAGCAATGCTCAGATCTTTATTGGATGTATTATCCGTTCCATCGAATGAAAAACTGTACTACGGCGGTAAAACCAATATGTTGAAGCAGCCGGAGTTTCAAGACATCGAGAAAATCCAAGGAATTATGAGTTTAATGGAAGAGGAAAATGATTTTTATCAAATTTTAAAACAAACGCCTTTTGGTATCCATGTAAAAATTGGTTCGGAAAATAAAATTCCGGTGATGGATGATTGCAGTTTAATTACGGCCACATATAGTATTGGTGAAGAACAGGTTGGGACGATTGCCATATTAGGCCCTAAGCGGATGCACTATTCCAAAGTGATTAGTCTGTTAAATCTACTGAGCAAAAATATGACCGTTACACTAACAAAGCTATATGATCTCTGA |
| *cotM* | ATGAATGGGAAAGATGAAAACGCCCCATTTGATTCCAAATTAATTGAAAACTGGTTTGAACAGTTTTTTCTTGATCCACTTACAACATACTTGGATAAAACAGTATTTCGTATTGACTTATTCGAAACTGAAAATGAATTTATTGTTGAGGCACTTTTACCGTCTTGTCAAAAGGAAAATATCCATGTTGCCGTTAACGGACAAGAACTTTCCATAAAAGTAAAGGATCACAAGAAAAATCATCCTCATTCCCACAATTATAAAATACGAACGGTTACGTTTCCCATGTCGATTGATGAACTCAAGATAGATACACGGTTTGAACATGATATACTTGAAATAAAATTATACAAAAAAGATAAATAA |
| *ctsR* | TTGAGAAATATTTCGGATATCATTGAGGAATATTTAAAGCAAATATTAGAGATGAGTGAAGAAGAAATATTAGAAATTAAACGCAGTGAGATTGCCAGTAAATTTCAATGTGTTCCTTCACAGATCAATTACGTTATTAATACAAGATTTACCCTTGAACGCGGCTATTTAGTTGAGAGTAAACGTGGCGGTGGCGGATATATTCGGATTATTAAAGTTAGACCGAATAGTAAGAAACACCTTATCGACCAATTGTTTGCGATCATTGGTAATCAAATTCCTCAATCTTCTGCTACAGATATCATAAATCGATTACGAAAAGAAGGAGTTATTAATGAACGTGAAGAAAAAATAATGTTAAGTGCACTGGATCGTTCAGTAATTTATTTAGACTTGCCCGAACGAGATTTAATAAGGGCAAGAATTTTAAAAGCAATGTTGTTAACTCTAAAGTATGAATAA |
| *hsp20A* | GATGAACAATTACCGGAAAAAAAGGACAGCCATTCAAATGAAGCTTCTCCATTTTTTCATATTTTTCAGGACCGGCCATTTCGTAATATTTTGGAGACCATTGATCAATTTTTTCAAAACTCCAATTTAAAACCGGGTTTCAAAGTCAATGTTCAAGAAGATGTTGATAAATACATCATATATGCAGAATTACCCGGGGTAAAGAAAAATCAAATTGACATCCATATTTTGGAGAGAAGCATTACCATCACTGTGAATCGTTCAGAAAGTGAGACAATTCAAAATGATAAAGCTAAATCATATACGAAGATACACGAATATGAAAAAGCCAGCCGGACAATTCCTTTTTATCATCCAATTCAATCAAAAAACGCCAAAGCCAAATACCGTGACGGGCTATTGACGATCATCCTGCCAAAAAAATCCGGGAAGAAACTGAATTTAGAAGATTGA |
| *hslR* | ATGAGGTTAGATAAATTTTTAAAAGTATCCCGATTGATAAAAAGGCGAACTTTGGCAAAAGAAGTAGCGGATCAGGGAAGAATTTTAATCAATGGAAAAACAGCTAAAGCAAGTTCCGAAGTCAATGTCGGGGATGAATTAGAAATCAGATTTGGACAAAGACTTATGACCGTGAAGATTGAAAAATTGCAAGAAACAACGCGAAAAGAAGATGCGGATTCAATGTATACCGTTATGAAAGATGAGCGGATAAATCAAGAAAACTAA |
| *hsp20B* | ATGGCATTAATACCTTATGATCCATTTAGACAATTAACTAACATTAGGAGAGATTTTGACAGATTATTCAATGATTTCCCTTTTCATTTAGACAATGATATGAATCATTTGGGGAATATAAGAGTGGATGTTCATGAAACAGATAAAGAGGTAGTAGCAACTTGTGACATTCCAGGCTTACAGAGTAAAGAAGATGTAAACATTGATATTGAAAATAATGTATTGAGAATTAGTGGCTCAATTAACAGAACTAATGAGATTAAAGAAGAGAATATGCATAGGAGAGAAAGATACACAGGTAGCTTTCACAGAGCTATTACACTACCAACTCCTGTTTCTGAGGAAGGTATAAAGGCTTCTTATAAGAATGGTGTACTAGAAGTTATCATGCCTAAACAAACTCAAACAAATAAAAAGAGAATTGATGTGGATTTCCATTAA |

Table S3 Summary of mass spectrometry data for flagellin and 30S ribosomal protein S4.

| Accession | Description | Gene | Mw | Coverage | Peptides | PSMs | Unique Peptides | Score |
| --- | --- | --- | --- | --- | --- | --- | --- | --- |
| WP_108898720.1 | flagellin | *filC* | 30.1 | 49% | 11 | 48 | 11 | 143.91 |
| WP_041847920.1 | 30S ribosomal protein S4 | *rpsD* | 23.1 | 48% | 10 | 21 | 10 | 64.58 |
